# Supplementary material for: B cell lineage reconstitution underlies CAR-T cell therapeutic efficacy in patients with refractory myasthenia gravis
Source: EMBO Mol Med. 2024 Feb 26;16(4):21. doi: 10.1038/s44321-024-00043-z (PMC11018773; doi:10.1038/s44321-024-00043-z)
Supplement: Supplementary file 8 — Expanded View Figures [file 44321_2024_43_MOESM8_ESM.pdf]

Expanded View Figures

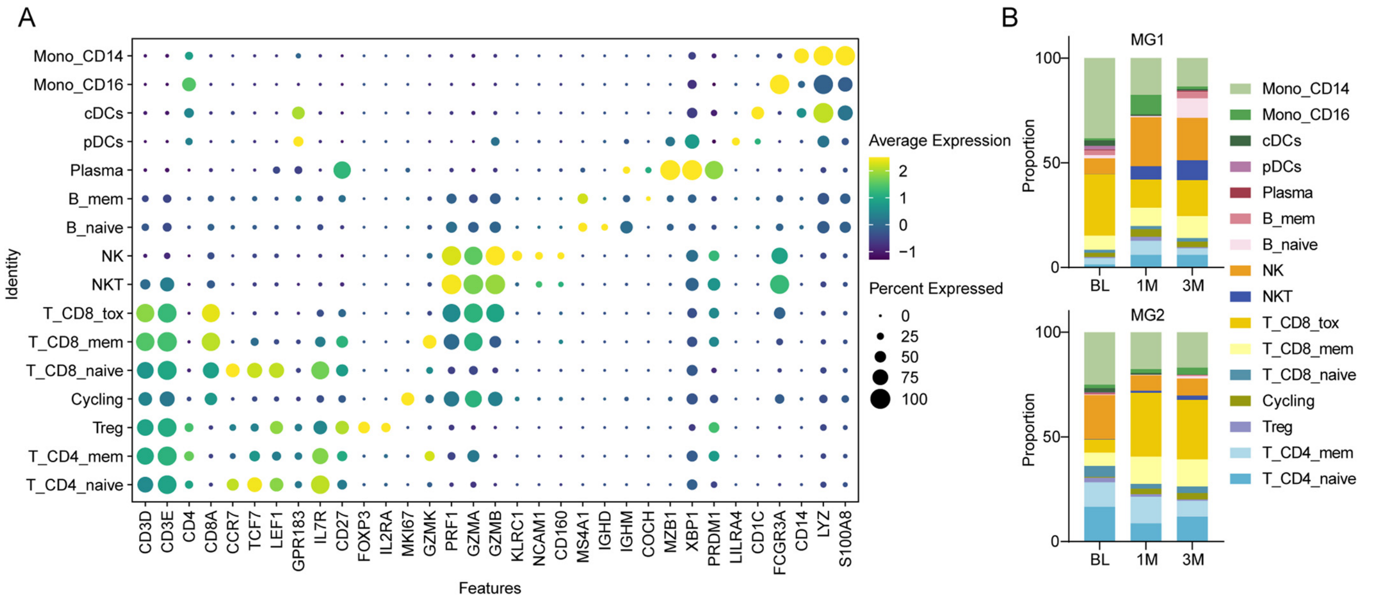

Figure EV1. Additional single-cell transcriptional features of patients with myasthenia gravis treated with CAR-BCMA T-cell therapy.

(**A**) Dot plot showing cell clusters denoted by gene expression of known markers. (**B**) Bar plots showing the frequency of cell subsets in individual patient at indicated time points.

© The Author(s)

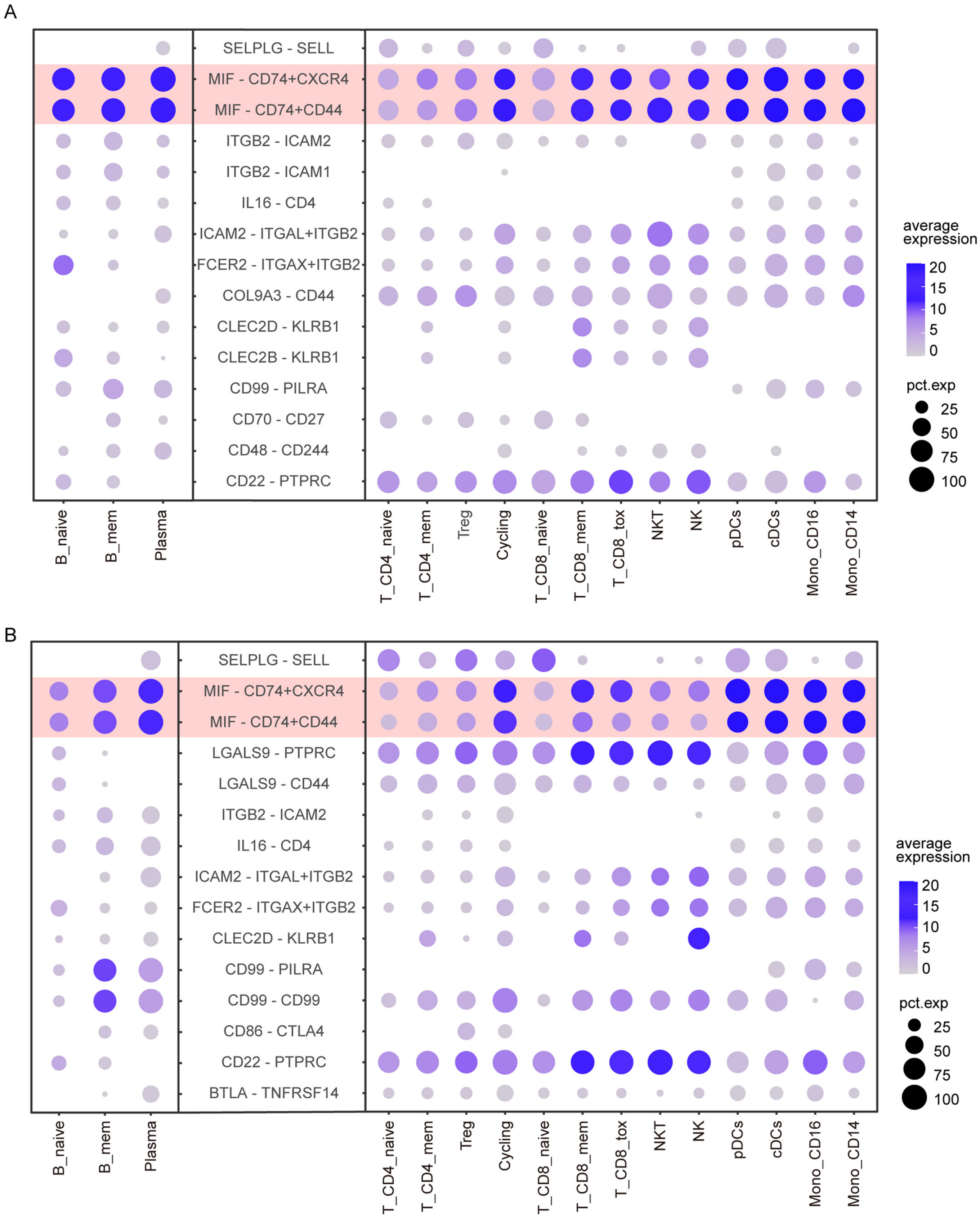
A
SELPLG - SELL
MIF - CD74+CXCR4
MIF - CD74+CD44
ITGB2 - ICAM2
ITGB2 - ICAM1
IL16 - CD4
ICAM2 - ITGAL+ITGB2
FCER2 - ITGAX+ITGB2
COL9A3 - CD44
CLEC2D - KLRB1
CLEC2B - KLRB1
CD99 - PILRA
CD70 - CD27
CD48 - CD244
CD22 - PTPRC
B_naive
B_mem
Plasma
T_CD4_naive
T_CD4_mem
Treg
Cycling
T_CD8_naive
T_CD8_mem
T_CD8_tox
NKT
NK
pDCs
cDCs
Mono_CD16
Mono_CD14
average expression
20
15
10
5
0
pct.exp
25
50
75
100
B
SELPLG - SELL
MIF - CD74+CXCR4
MIF - CD74+CD44
LGALS9 - PTPRC
LGALS9 - CD44
ITGB2 - ICAM2
IL16 - CD4
ICAM2 - ITGAL+ITGB2
FCER2 - ITGAX+ITGB2
CLEC2D - KLRB1
CD99 - PILRA
CD99 - CD99
CD86 - CTLA4
CD22 - PTPRC
BTLA - TNFRSF14
B_naive
B_mem
Plasma
T_CD4_naive
T_CD4_mem
Treg
Cycling
T_CD8_naive
T_CD8_mem
T_CD8_tox
NKT
NK
pDCs
cDCs
Mono_CD16
Mono_CD14
average expression
20
15
10
5
0
pct.exp
25
50
75
100

 © The Author(s)

Figure EV2. Additional ligand-receptor interaction analysis between immune cells.

Gene expression dot plot of ligand-receptor expression. Left, expression in B-cell subsets; right, expression in other immune cell types. Shown are the Top15 interactions between B-cell subsets and other immune cell types with communication probabilities in (**A**) MG-1, (**B**) MG-2 at baseline.

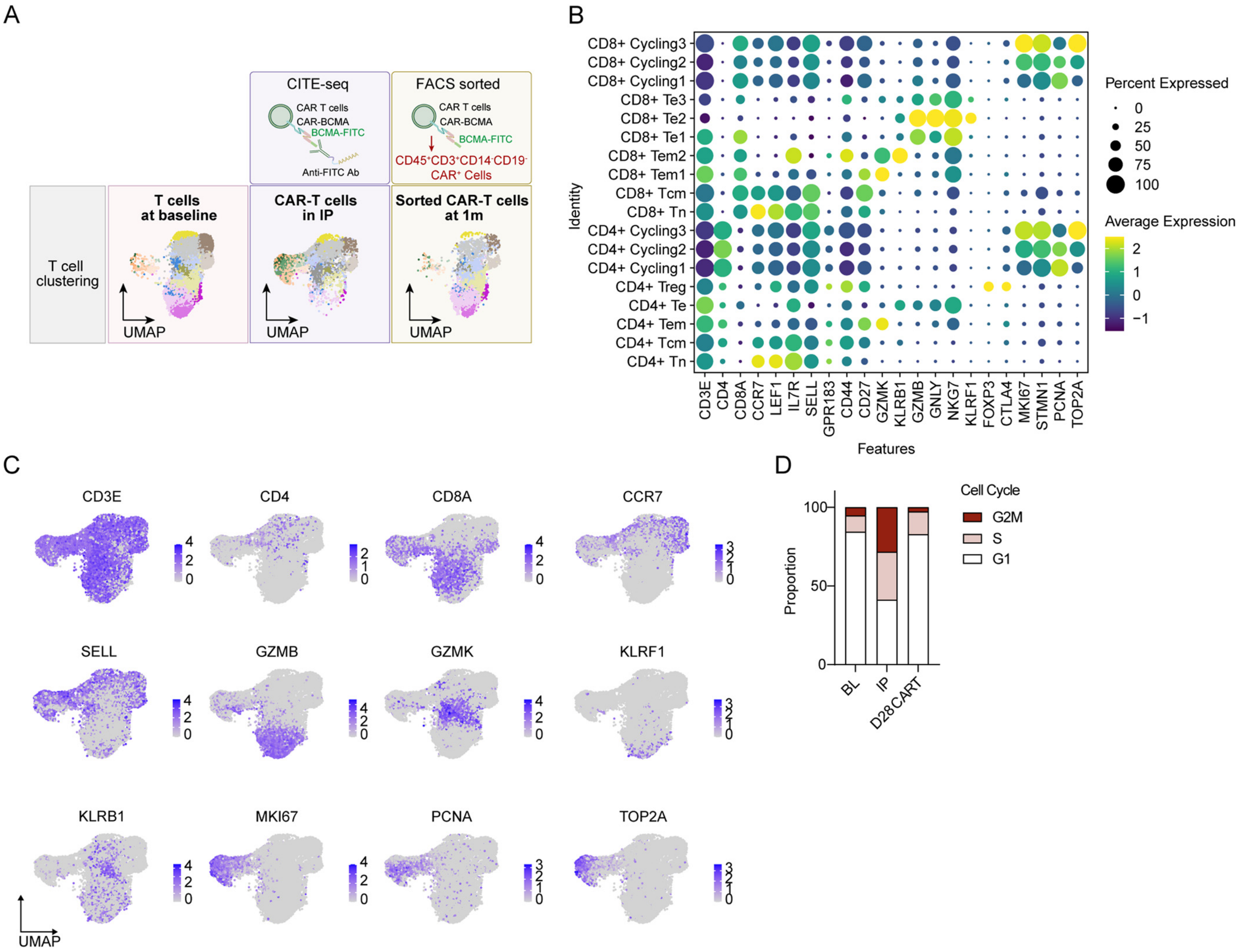

Figure EV3. Expression of canonical markers identifies behavior of CAR-T cells in patients with myasthenia gravis.

(**A**) Schematic illustration of cellular indexing of transcriptomes and epitopes by sequencing (CITE-seq) strategy used to detect the CAR on the T-cell surface, and flow cytometry to sort CAR-T cells in vivo at 1 month post infusion (see "Methods" for details). UMAP plots of T cells colored by different cell cluster and clone size. (**B**) Dot plots showing gene expression of known markers for T-cell and CAR T-cell subclusters. (**C**) UMAP plots indicating RNA expression of *CD3E, CD4, CD8A, CCR7, SELL, GZMB, GZMK, KLRF1, KLRB1, TOP2A, MKI67*, and *PCNA*. (**D**) Bar plots showing the frequency of cells in S phase or G2/M phase by cell cycle scoring (see "Methods" for details).

© The Author(s)

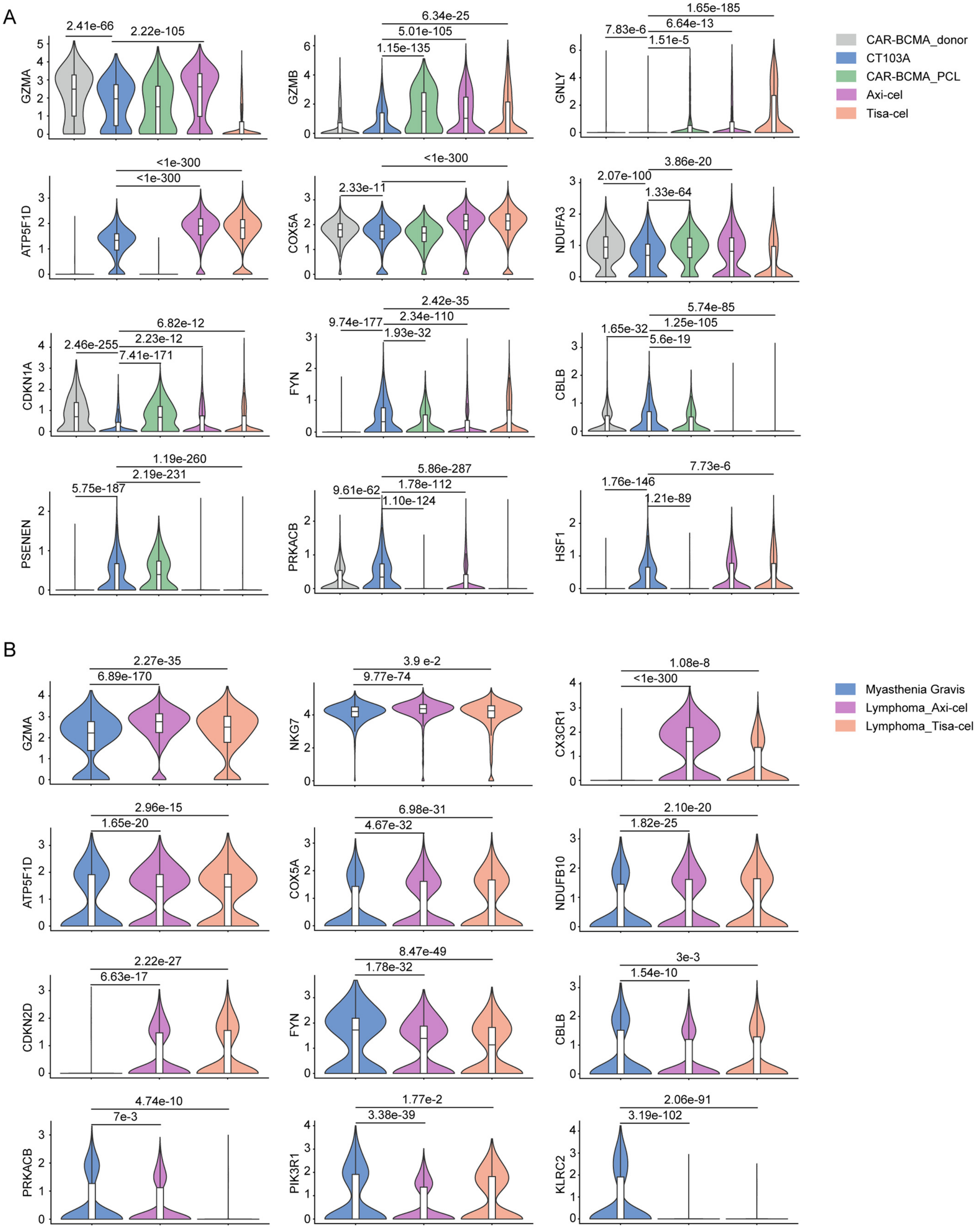
A
2.41e-66
2.22e-105
GZMA
6.34e-25
5.01e-105
1.15e-135
GZMB
1.65e-185
7.83e-6
6.64e-13
1.51e-5
GNLY
CAR-BCMA_donor
CT103A
CAR-BCMA_PCL
Axi-cel
Tisa-cel
<1e-300
<1e-300
ATP5F1D
<1e-300
2.33e-11
COX5A
3.86e-20
2.07e-100
1.33e-64
NDUFA3
6.82e-12
2.46e-255
2.23e-12
7.41e-171
CDKN1A
2.42e-35
9.74e-177
2.34e-110
1.93e-32
FYN
5.74e-85
1.65e-32
1.25e-105
5.6e-19
CBLB
1.19e-260
2.19e-231
5.75e-187
PSENEN
5.86e-287
9.61e-62
1.78e-112
1.10e-124
PRKACB
7.73e-6
1.76e-146
1.21e-89
HSF1
B
2.27e-35
6.89e-170
GZMA
3.9 e-2
9.77e-74
NKG7
1.08e-8
<1e-300
CX3CR1
Myasthenia Gravis
Lymphoma_Axi-cel
Lymphoma_Tisa-cel
2.96e-15
1.65e-20
ATP5F1D
6.98e-31
4.67e-32
COX5A
2.10e-20
1.82e-25
NDUFB10
2.22e-27
6.63e-17
CDKN2D
8.47e-49
1.78e-32
FYN
3e-3
1.54e-10
CBLB
4.74e-10
7e-3
PRKACB
1.77e-2
3.38e-39
PIK3R1
2.06e-91
3.19e-102
KLRC2

Figure EV4. Expression of indicated genes identifies characteristics of CD8 + Te cells (Baseline) and CD8+ cycling CAR-T cells (IP) in patients with myasthenia gravis.

(**A**) Violin plots illustrating indicated genes in Fig. 5B, and representing the distribution of expression across each product. CT103A, $N = 4044$ cells; CAR-BCMA_donor, $N = 4739$ cells; CAR-BCMA_PCL, $N = 2111$ cells; Axi-cel, $N = 11{,}392$ cells; Tisa-cel, $N = 16{,}348$ cells. Boxes show median, Q1 and Q3 quartiles and whiskers up to 1.5× interquartile range. Pairwise comparisons were performed using a two-sided Wilcoxon rank-sum test with a Bonferroni correction. (**B**) Violin plots illustrating indicated genes in Fig. 5C, and representing the distribution of expression across each cohort. Myasthenia Gravis, $N = 2898$ cells; Lymphoma_Axi-cel, $N = 7123$ cells; Lymphoma_Tisa-cel, $N = 4857$ cells. Boxes show median, Q1 and Q3 quartiles and whiskers up to 1.5× interquartile range. Pairwise comparisons were performed using a two-sided Wilcoxon rank-sum test with a Bonferroni correction.

 © The Author(s)
